# Understanding the Rift Valley fever exposure risk: A comparative perspective from a multi-country study in East and Central Africa, 2021-24

**DOI:** 10.1371/journal.pntd.0014082

**Published:** 2026-03-10

**Authors:** Luciana Lepore, Raymond Odinoh, Jeanette Dawa, Silvia Situma, Luke Nyakarahuka, Sheila Makiala, Hervé Viala, Christian Ifufa, Marie-Anne Kavira Muhindo, John Kayiwa, Nicholas Awor, Noella Mulopo-Mukanya, Alex Tumusiime, Anne Hauner, Jackson Kyondo, Stijn Rogé, Carolyne Nasimiyu, Steve Kisembo, Annemarion Namutebi, David Odong, Hugo Kavunga-Membo, Julius Lutwama, Ézéchiel Bushu Mulinda, Kevin K. Ariën, Daniel Mukadi-Bamuleka, Deo Ndumu, Jean-Jacques Muyembe Tamfum, Robert F. Breiman, Barnabas Bakamutumaho, Kariuki Njenga, Justin Masumu, Veerle Vanlerberghe

**Affiliations:** 1 Department of Public Health, Institute of Tropical Medicine, Antwerp, Belgium; 2 Washington State University Global Health Program, Nairobi, Kenya; 3 Center for Research in Emerging Infectious Diseases-East and Central Africa, Nairobi Kenya; 4 Center for Epidemiological Modelling and Analysis, University of Nairobi, Nairobi, Kenya; 5 Department of Animal Science, Pwani University, Kilifi, Kenya; 6 Department of Biosecurity, Ecosystems and Veterinary Public Health, College of Veterinary Medicine, Animal Resources, and Biosecurity, Makerere University, Kampala, Uganda; 7 Viral Hemorrhagic Fever Surveillance Program, Uganda Virus Research Institute, Entebbe, Uganda; 8 Department of Global Health, Rollins School of Public Health, Emory University, Atlanta, GeorgiaUnited States of America; 9 Department of Virology, Institut National de Recherche Biomédicale, INRB, Kinshasa, Democratic Republic of the Congo; 10 Department of Medical Biology, Faculty of Medicine, University of Kinshasa, Kinshasa, Democratic Republic of the Congo; 11 Laboratoire Rodolphe Mérieux INRB-Goma, Goma, Democratic Republic of the Congo; 12 Department of Epidemiology and Global Health, Institut National de Recherche Biomédicale, INRB, Kinshasa, Democratic Republic of the Congo; 13 Department of Arbovirology, Emerging and Re-emerging Infectious Diseases, Uganda Virus Research Institute, Entebbe, Uganda; 14 Department of Biomedical Sciences, Institute of Tropical Medicine, Antwerp, Belgium; 15 DRC Office, Institute of Tropical Medicine, Antwerp, Belgium; 16 Hôpital Général de Référence Virunga, Goma, Democratic Republic of the Congo; 17 Kabale Regional Referral Hospital, Kabale, Uganda; 18 Laboratoire Vétérinaire de Goma, Labovet, Goma, Democratic Republic of the Congo; 19 Service of Microbiology, Department of Medical Biology, Kinshasa University Hospital, Faculty of Medicine, University of Kinshasa, Kinshasa, Democratic Republic of the Congo; 20 Ministry of Agriculture, Animal Industry and Fisheries, Entebbe, Uganda; 21 Institut National de Recherche Biomédicale, INRB, Kinshasa, Democratic Republic of the Congo; 22 Infectious Diseases and Oncology Research Institute, University of the Witwatersrand, Johannesburg, South Africa; 23 Paul G. Allen School for Global Health, Washington State University, Pullman, Washington, United States of America; 24 Cellule d’appui à la Recherche, Institut National de Recherche Biomédicale, INRB, Kinshasa, Democratic Republic of the Congo; Makerere University, UGANDA

## Abstract

Rift Valley fever (RVF) is a concern in East and Central Africa, particularly following periods of heavy rainfall and flooding. However no human outbreaks have been reported in the Democratic Republic of the Congo (DRC). To assess whether this reflects a true absence of virus circulation, we estimated RVF seroprevalence in Goma (eastern DRC) and examined context-specific risk factors, comparing the findings with data from outbreak-prone countries. A two-year longitudinal study, across six health facilities in DRC, Kenya and Uganda, enrolled febrile subjects aged ≥10 years. Human serum samples were analyzed for RVF virus and anti-RVF antibodies. Demographic, behavioral, occupational and environmental factors were evaluated. 4,806 participants were enrolled: 1,370 (28.5%) DRC, 1,468 (30.6%) Kenya and 1,968 (40.9%) Uganda. 253 participants (5.3%) tested positive for RVF by serological and/or molecular assays: 19 (1.4%) DRC, 29 (2.0%) Kenya and 205 (10.4%) Uganda (p < 0.001). Only in Uganda, subjects tested positive for RVF virus by PCR (10 subjects, 0.5%). Occupations and activities involving contact with livestock, predominated in Kenya and Uganda, whereas handling of raw meat was most common in DRC. No specific occupations or activities were significantly associated with RVF exposure in DRC while several significant factors were identified for Kenya and Uganda. Multivariate analysis across all three countries showed that being from Uganda, male, over 20 years of age, employed as butcher or crop farmer and engaging in animal-related activities, were independently associated with RVF positivity, as was contact with sheep. Despite a prevailing sense that RVF transmission does not occur in DRC, we found a seroprevalence of 1.4%, comparable to Kenya where RVF is well documented. Further research targeting high-risk human and animal populations in DRC is warranted. A One Health approach will contribute to defining the ecology of local transmission of RVF in DRC.

## Author summary

Rift Valley fever (RVF) is a zoonotic arthropod-borne viral disease that threatens global health security. Although several outbreaks have been reported in East Africa and RVF virus circulation has previously been described in small ruminants and cattle in the Democratic Republic of Congo (DRC), to date there have been no reported outbreaks of RVF in humans in the DRC. We therefore investigated in Goma, eastern DRC, seroprevalence in humans and risk factors for exposure to RVF virus, and compared the results with those from neighbouring outbreak-prone countries, Kenya and Uganda. Despite a prevailing sense that RVF transmission does not occur in the DRC, we found a seroprevalence of 1.4%, similar to that observed in Kenya. No significant exposure factors were identified for the study population in the DRC alone, but joint analysis with Kenya and Uganda revealed several factors associated with previous exposure to the RVF virus, such as country and various types of contact with livestock. Further research targeting the most at-risk human and livestock populations in the DRC is needed to understand the underlying virus circulation mechanisms.

## Background

Rift Valley fever (RVF) is a zoonotic arthropod-borne viral disease affecting both humans and animals, mainly livestock. Since 2016, the RVF virus has been prioritised by the World Health Organisation (WHO) for urgent research and development of countermeasures aimed at preventing and controlling future outbreaks [1]. The scarcity of reliable countermeasures available to date against RVF continues to threaten global health security. The main reasons for RVF being a priority pathogen, are fourfold: the absence of efficacious antivirals and/or vaccines; the importance of cross-border dynamics associated with livestock trade and the displacement of people; the catastrophic direct and indirect social and economic effects, causing, among others, food insecurity and poverty [2]; and the estimation of increased wider geographic range for RVF virus in the future, based on climate change’s impact on RVF transmission, associated with heavy rainfall favoring mosquito propagation. Furthermore, guidelines and guidance documents on RVF diagnosis, prevention and treatment are scarce [3]. RVF surveillance is described as heterogeneous, incomplete and patchy, often triggered by outbreaks. Its activities are based on national viral hemorrhagic fever surveillance systems, as in Mauritania [4], or combining passive and active surveillance, as in Uganda [5]. RVF surveillance is occasionally integrated into One Health approaches such as the RVF action framework by the Food and Agriculture Organization [6] and the One Health strategic plan for the prevention and control of zoonotic diseases in Kenya [7]. This strategy aims at establishing active collaboration at the animal-human-ecosystem interface for better prevention and control of (re-)emerging zoonotic diseases. There is increasing evidence that RVF transmission may go undocumented, as probably occurred in Burundi prior to the explosive RVF outbreak in 2022 [8], and substantial undetected transmission of RVF virus occurs during inter-epidemic periods [9]. In Kenya, the continuous detection of low levels of anti-RVF IgG antibodies in humans and livestock indicates possible hotspots of virus circulation and maintenance during interepidemic periods [10].

Sub-Saharan Africa has historically been affected by RVF outbreaks, with Sudan, Somalia and Kenya among the countries where major outbreaks have occurred [11–13]. In these countries, the presence of permissive ecologies coupled with favorable climatic and environmental conditions, like El Niño, are key climatic drivers of epidemics [14,15]. Most RVF seroprevalence studies have been conducted in East and Central Africa (ECA), specifically in Kenya and Tanzania, with reported seroprevalence rates of 9.6% and 6.7%, respectively [16]. In more recent years, the spread of RVF has been a serious threat to public health in this region: an unexpected epidemic in 2018 spread beyond Kenya, South Sudan and Tanzania, also affecting Rwanda and Uganda suggesting changes in the dynamics of disease transmission [8]. Further evidence supports a change in the RVF epidemiology in Uganda and Kenya where an increased frequency of small RVF clusters has been identified in areas previously unaffected including highlands where there have been significant land use changes (rice cultivation, mining and irrigation), resulting in substantial water accumulation [5,17]. By contrast, the Democratic Republic of the Congo (DRC) stands out in the ECA region because of an absence of reported RVF outbreaks. However, studies have highlighted the circulation of the RVF virus in the country, in small ruminants [18] and cattle, especially in the eastern DRC (North Kivu and Ituri provinces) [19]. *Aedes* mosquitoes also tested positive for RVF virus in the Ndjili municipality (south-eastern Kinshasa, along the Ndjili River), where pig farming and agriculture are practiced [20]. Since 2000, the DRC has adopted an integrated disease surveillance and response strategy due to the emergence of multiple infectious disease outbreaks. Nevertheless, the surveillance system seems to be more outbreak-focused rather than on routine case-based surveillance [21]. This could partly explain the absence of reports of sporadically occurring RVF in humans, despite permissive ecologies in the DRC. Additionally, the eastern DRC is bordering countries (Uganda, Rwanda and Burundi) with a demonstrated RVF transmission, including outbreaks. Considering the evolving epidemiology of RVF and its expansion within ECA, eastern DRC could be at risk of an outbreak. Thus, estimating seroprevalence and understanding context-specific risk factors for RVF exposure in the DRC are paramount, using neighboring countries with known RVF transmission as reference to inform protocols for case detection and epidemic preparedness [16]. The objective of our study was to i) understand the transmission of RVF based on seroprevalence and risk factors in Goma, and ii) to compare the epidemiological profile of RVF cases with neighboring areas of Kenya and Uganda. This was realized as part of a multi-country initiative of the Center for Research on Emerging Infectious Diseases - East and Central Africa (CREID-ECA).

## Methods

### Ethics statement

The study protocol was reviewed and approved by the ethics review committees of each country including the School of Public Health, University of Kinshasa (UNIKIN), DRC (ref. ESP/CE/122/2021, ESP/CE/086/2022 and ESP/CE/108/2023); Kenya Medical Research Institute (KEMRI) - Research Ethics Committee (ref. SERU 4169) licensed by the National Commission for Science, Technology and Innovation (NACOSTI: License No: NACOSTI/P/24/38396); Uganda Virus Research Institute (UVRI) (ref. GC/127/849) and Uganda National Council of Science and Technology (ref. HS1713ES). Reliance Agreements were provided by the Washington State University. The study was further approved in Belgium by the Institutional Review Board of the Institute of Tropical Medicine Antwerp (ref. 1462/21) and the Ethical Committee of the Antwerp University Hospital UZA (ref. B3002021000105). The study protocol was registered on the ClinicalTrials.gov, Identifier: NCT05139524.

Informed written consent for participation was requested from all participants and was provided in the respective native language; with minors, written consent was provided by their respective parents/guardians. Assent forms for minors aged 10–17 years old were also collected.

### Study design and setting

**Fig 1:**
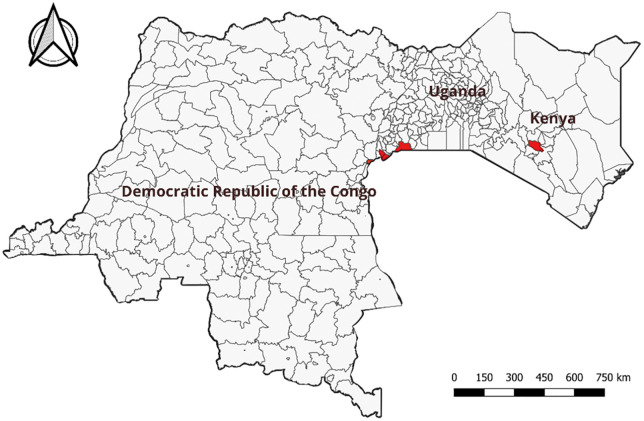
Study sites (in red) in the Democratic Republic of the Congo (1 site), Kenya (2 sites) and Uganda (3 sites), 2021-2024. Map created using QGIS version 3.28. Base maps: Administrative boundaries from Humanitarian Data Exchange for Democratic Republic of Congo (https://data.humdata.org/dataset/cod-ab-cod), Kenya (https://data.humdata.org/dataset/cod-ab-ken), and Uganda (https://data.humdata.org/dataset/cod-ab-uga). All datasets licensed under CC BY-IGO.

We conducted a prospective cohort study between October 2021 and February 2024. Study sites were selected within the ECA: Kenya and Uganda, both affected by previous RVF outbreaks in animals and humans; and the DRC, where no RVF related hemorrhagic fever has been documented in humans to date. We involved six health facilities across the ECA region: one in the Eastern-DRC, the Virunga Referral General Hospital in Goma; two in central Kenya, Kigetuini Dispensary and Kandara sub Country Hospital; and three in south-western Uganda, Rwekubo Health Center, Kabale Regional Referral Hospital and Hamurwa Health Center (Fig 1).

In the DRC, the study was conducted in Goma, the capital and largest city of the province of North Kivu in the eastern part of DRC. Facing Lake Kivu and home of the Virunga Park, Goma is characterized by a predominantly volcanic terrain and a tropical savannah climate tempered by its altitude of about 1500 meters. The environment of the city is typically urban, although its economy relies mainly on agriculture and therefore maintaining tight connections with its rural surroundings. Over the years the city has increasingly become a transit hub for trade and has served as a major site for internally displaced people, being afflicted by a long conflict and instability. In Uganda, the study was conducted in the Kabale, Isingiro and Rubanda districts, in the rural southwestern part of the country, characterized with both low and highland terrain, where cattle and goat farming and small-scale agriculture are the main activities [22]. The study sites in Kenya were located in Murang’a County, within the central highlands where intermittent cases of RVF have been described among animals and humans [23].

### Study population and study variables

We prospectively included subjects aged ≥10 years who presented for an outpatient consultation at the selected health facilities. Consenting subjects with a history of fever in the last four weeks (body temperature ≥37.5°C) and/or unexplained bleeding and/or unknown infectious illness treated for >7 days and unresponsive to treatment, were enrolled. Given the potential for co-infection and the also vector-borne transmission route for RVF, cases diagnosed with malaria were included up to a maximum of 20% of the study sample. Pregnant women were included based on the likelihood to be infected with RVF virus and the abortive effects reported in livestock, but which are not yet well described/understood in humans [24]. We excluded patients who were diagnosed with a urinary tract infection or COVID-19 or another condition (other than malaria) that explained their febrile illness. In addition, patients were excluded from the study if they had been hospitalized for more than 48 hours during the previous 14 days. We collected socio-demographic, clinical, household characteristics, behavioral and knowledge information. All data were de-identified and collected by electronic questionnaires on REDCap [25]. For each participant, a venous blood sample was obtained in BD Vacutainer SST and EDTA tubes for the detection of RVF virus ribonucleic acid (RNA) and anti-RVF antibodies. The estimated sample size over the 2 years was 1600 participants for the single study site in the DRC and 707 participants per study site in Kenya and Uganda. The sample size was calculated to detect a seasonal difference of acute cases, estimated at 3% in the rainy season and 1% in the dry season, with a confidence level of 95%, a power of 80% and a precision of 2% [26,27].

### Laboratory procedures

Blood samples collected at each health facility were transported to the respective laboratories for centrifugation and serum aliquoting in cryotubes. The aliquots were stored at -80 °C for further testing. Malaria testing was performed on-site in accordance with existing Ministry of Health guidelines of the respective countries and based on rapid diagnostic tests (RDTs) detecting parasite lactate dehydrogenase (pLDH) or histidine-rich protein 2 (HRP2) RDT and/or via thick blood smear. Conversely, laboratory analysis for RVF infection was performed within the respective country-reference laboratories and following standardized protocols developed within the project and jointly adopted by the three participating countries, as detailed below.

### RVF virus molecular analyses

All samples collected at enrolment were tested by reverse transcription quantitative polymerase chain reaction (RT-qPCR) to detect RVF virus RNA. The QIAamp Viral RNA Mini kit (Qiagen, Germany) was used for nucleic acid extraction according to the manufacturer’s instructions. A one step real-time RT (reverse-transcription)-PCR was performed using the iTaq Universal Probes One-Step Kit (Biorad) on a CFX96 Real-Time PCR Detection System (Bio-Rad Laboratories, USA). For a total volume of 25µL, 20µL of master mix (12.5µL of iTaq Universal Buffer, 0.5µL of iScript RT, corresponding concentrations of primers and probes, with nuclease-free H2O) and 5µL of RNA were added. RVF virus-PCRs from previously published protocols [28,29] were used to detect samples with RVFV-RNA. The RT-PCR program consisted of four steps, with steps 3 and 4 repeated 50 times: step 1 at 50°C for 600 sec, step 2 at 95°C for 300 sec, step 3 at 95°C for 10 sec and step 4 at 57°C for 30 sec. For baseline subtracted curve fit was set by the CFX96 data analysis program and single threshold was used. Samples that tested positive for RVF virus in PCR were subsequently inactivated by heat. PCR assays performed in Kenya and Uganda, were described elsewhere [23,30].

### RVF virus serological analyses

All collected samples were tested for anti-RVF virus antibodies. Serological testing consisted of an initial ELISA test for total RVF virus antibodies, followed, if positive, by an ELISA for RVF virus-IgM detection. For the purpose of our study, commercially available ELISA kits were used; multi-species RVFV competitive enzyme-linked immunosorbent commercial assay (C-ELISA) from IDvet (Grabels, France) and the Abbexa IgM capture ELISA (Grables, France) [23]. Kits were used according to the production company’s recommended procedures.

### Statistical analysis

#### Descriptive statistics.

De-identified data from the six study sites were combined and analyzed using R statistical software (version 4.4.1; R Foundation for Statistical Computing, Vienna, Austria). Results were summarized using descriptive statistics with continuous variables reported as median with interquartile ranges (IQRs), and categorical variables as frequences and percentages. The main outcome was RVF positivity defined as a participant testing positive on at least one RVF-specific diagnostic assay (PCR and/or total antibodies and/or IgM).

#### Handling of missing data.

Missing data (<0.3%) were addressed using random imputation, whereby missing values were replaced with randomly selected observed values from the same variable.

#### Bivariate analysis.

Categorical variables were compared using the chi-square and Fisher’s exact test, as appropriate, with statistical significance set at p < 0.05. Bivariate analyses (crude Odds ratios) were conducted to assess associations between each independent variable and RVF positivity.

#### Multivariable modeling.

Variables that had a p < 0.1 in bivariate analyses -including the global p-value for categorical variables with more than 2 levels- were included in a multivariable logistic regression model. Variable selection was conducted using a backward elimination method, based on Akaike Information Criterion (AIC) [31], to obtain the most parsimonious model. Model fit was evaluated using the Hosmer-Lemeshow goodness-of-fit test. Adjusted odds ratios (aORs) and 95% confidence intervals (CIs) were reported, with statistical significance defined as p < 0.05.

## Results

A total of 4,806 subjects were enrolled over the overall 2-year period: 1,370 (28.5%) from the DRC, 1,468 (30.6%) from Kenya and 1,968 (40.9%) from Uganda.

Socio-demographic data and key features at enrollment are depicted in Table 1. Overall participants median age was 31 years old (IQR: 22–44) with a slightly older population in Kenya (median age 35, IQR: 24–47), compared to the DRC (median age 29, IQR: 23–41) and Uganda (median age 30, IQR 22–41). Young adults between 21 and 40 years old were the most represented (51.9%) along with 2,763 women (57.5%). More than half of the DRC population had attained an educational level beyond completed primary education (51.2%). Nevertheless, the DRC also exhibited the highest proportions of unemployed individuals (42.5%), along with skilled workers (12.3%), healthcare workers (7.1%) and students (17.0%). In contrast, the other two countries reported lower levels of education, especially in Uganda, where low schooling reached as high as 74.4%. In Kenya and Uganda, the most common occupations were crop and animal farmers, in Kenya 27.2% and 24.2%, respectively, while in Uganda 60.8% and 15.4%, respectively. In the DRC, those occupations were by far less frequently reported (2.7% and 0.8%, respectively).

**Table 1:** Main socio-demographic, inclusion assessment and laboratory features, for the overall study population (n = 4806) and by country, in the DRC (n = 1370), Kenya (n = 1468) and Uganda (n = 1968), 2021-2024.

	OVERALL N (col%)	DRC N (col%)	KENYA N (col%)	UGANDA N (col%)
Enrolments	4,806 (100.0)	1,370 (28.5)	1,468 (30.6)	1,968 (40.9)

**SOCIO-DEMOGRAPHIC FEATURES**				
Age group				
10-20 years	925 (19.2)	243 (17.7)	255 (17.4)	427 (21.7)
21-40 years	2,494 (51.9)	775 (65.6)	679 (46.2)	1,040 (52.8)
Above 40 years old	1,387 (28.9)	352 (25.7)	534 (36.4)	501 (25.5)
Gender, Female	2,763 (57.5)	939 (68.5)	734 (50.0)	1,090 (55.4)
Education				
High schooling	1,761 (36.6)	702 (51.2)	555 (37.8)	504 (25.6)
Low schooling	3,045 (63.4)	668 (48.8)	913 (62.2)	1,464 (74.4)
Occupation				
Healthcare worker	182 (3.8)	97 (7.1)	17 (1.2)	68 (3.5)
Skilled labour	462 (9.6)	169 (12.3)	144 (9.8)	149 (7.6)
Unskilled labour	529 (11.0)	111 (8.1)	280 (19.1)	138 (7.0)
Animal farmer	668 (13.9)	11 (0.8)	355 (24.2)	302 (15.4)
Crop farmer	1,633 (34.0)	37 (2.7)	400 (27.2)	1,196 (60.8)
Butcher/slaughterhouse worker	33 (0.7)	3 (0.2)	15 (1.0)	15 (0.8)
Student	618 (12.9)	233 (17.0)	155 (10.6)	230 (11.7)
None	1009 (21.0)	582 (42.5)	338 (23.0)	89 (4.5)

**ASSESSMENT AT INCLUSION**				
Reported fever	4,521 (94.1)	1,334 (97.4)	1,390 (94.7)	1,797 (91.3)
Reported fever onset ≤7 days	3,426 (71.3)	1,187 (86.6)	750 (51.1)	1,489 (75.7)
Fever at inclusion (BT ≥ 37.5°C)	2,896 (60.3)	1,073 (78.3)	338 (23.0)	1,485 (75.5)
Bleeding	437 (9.1)	40 (2.9)	29 (2.0)	368 (18.7)
Similar disease in family*	577 (12.0)	77 (5.6)	117 (8.0)	383 (19.5)
Similar disease in community*	362 (7.5)	21 (1.5)	73 (5.0)	268 (13.6)
Unusual illness in community	271 (5.6)	4 (0.3)	26 (1.8)	241 (12.2)
Unexplained death in community	216 (4.5)	3 (0.2)	11 (0.7)	202 (10.3)
Abortions in herds	303 (6.3)	3 (0.2)	122 (8.3)	178 (9.0)
Unexplained death in herds	469 (9.8)	2 (0.1)	158 (10.8)	309 (15.7)
Unexplained deaths in wild animals	30 (0.6)	0 (0.0)	14 (0.9)	16 (0.8)

**LABORATORY TEST**				
RVF positivity (molecular/serological)	253 (5.3)	19 (1.4)	29 (2.0)	205 (10.4)
RVF virus PCR, positive	10 (0.2)	0 (0.0)	0 (0.0)	10 (0.5)
RVF virus IgM, positive	95 (2.0)	1 (0.1)	0 (0.0)	94 (4.8)
RVF virus Ig tot, positive	241 (5.0)	19 (1.4)	29 (2.0)	193 (9.8)
At least 1 malaria test performed	3,055 (63.6)	1,346 (98.2)	105 (7.1)	1,604 (81.5)
Positive n (%) tested	553 (18.1)	235 (17.5)	6 (5.7)	312 (19.5)
Positive n (%) tot pop	553 (11.5)	235 (17.1)	6 (0.4)	312 (15.8)

BT, body temperature; col, column; DRC, Democratic republic of the Congo; Ig, immunoglobuline; N, number; PCR, polymerase chain reaction; pop, population; RDT, rapid diagnostic test; RVF Rift Valley fever; tot, total; *In the last 2 months.

Nearly all participants reported fever (94.1%) which was the predominant inclusion criterion met, and 3,426 cases (71.3%) reported fever onset during the 7 days prior to study inclusion. The objective measuring of temperature at inclusion resulted in 2,896 (60.3%) subjects with a body temperature greater or equal to 37.5°C, representing over two thirds of the populations enrolled in the DRC (78.3%) and Uganda (75.5%). Bleeding signs were reported in 9.1% of the overall population, with a prominent detection, however, in Uganda, as high as 18.7% while in the DRC and Kenya this was reported in <3%. A certain proportion of the overall enrolled population reported similar health conditions within their family or community, as well as cases of unexplained deaths in the community. These occurrences were consistently less frequent in the DRC (all ≤ 5%), whereas Uganda showed markedly higher levels across all such indicators (all > 10%).

### Diagnostic investigation

Overall, a total number of 253 participants (5.3%) tested positive for RVF by serology and/or molecular testing with a significant difference between countries with 19/1370 (1.4%) subjects in DRC, 29/1468 (2.0%) subjects in Kenya and 205/1,968 (10.4%) subjects in Uganda, p < 0.001 (Table 1).

More specifically, in the Ugandan cohort alone, 10 subjects (0.5%) tested positive for the RVF virus-PCR, whereas no PCR was found positive in the Kenyan and the DRC study populations. Anti-RVF virus IgMs were detected in 94 subjects from Uganda (4.8%), in only 1 from the DRC (0.1%), and none in Kenya. Total anti-RVF virus antibodies were positive in 193 subjects in Uganda (9.8%), 29 in Kenya (2.0%), and 19 in the DRC (1,4%) (Table 1).

In relation to enrolled cases with suspected malaria, that were limited to a maximum of 20% of the study population as per study protocol, more than half of the population included (63.6%) received a malaria test by RDT and/or thick blood smear for microscopy. Positivity to at least one of the two malaria tests was recorded in 11.5% of the overall study population with higher prevalences in the DRC (17.1%) and Uganda (15.8%) compared to Kenya (0.4%) (Table 1).

### Assessment of exposure factors to RVF virus

To explore RVF virus exposure factors of our participants residing in at-risk areas, a combination of occupational, animal-related daily activities and environmental factors influencing mosquito abundance were investigated and compared among the three study cohorts.

### Animal exposure

**Fig 2:**
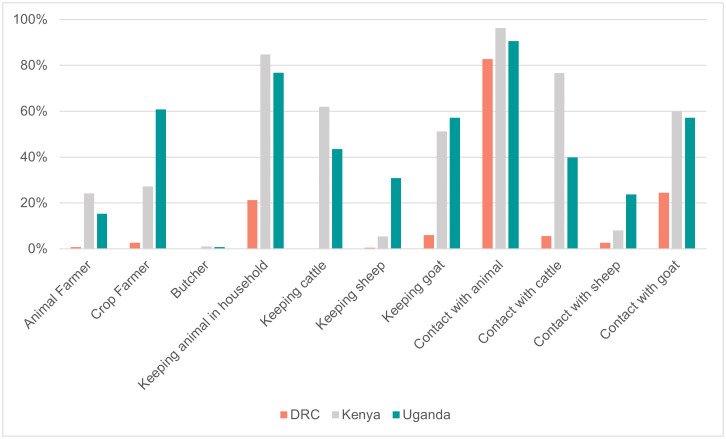
Contact with animals and livestock species reported by study participants, in the Democratic Republic of the Congo (DRC) (n = 1370), Kenya (n = 1468) and Uganda (n = 1968), 2021-2024.

**Fig 3:**
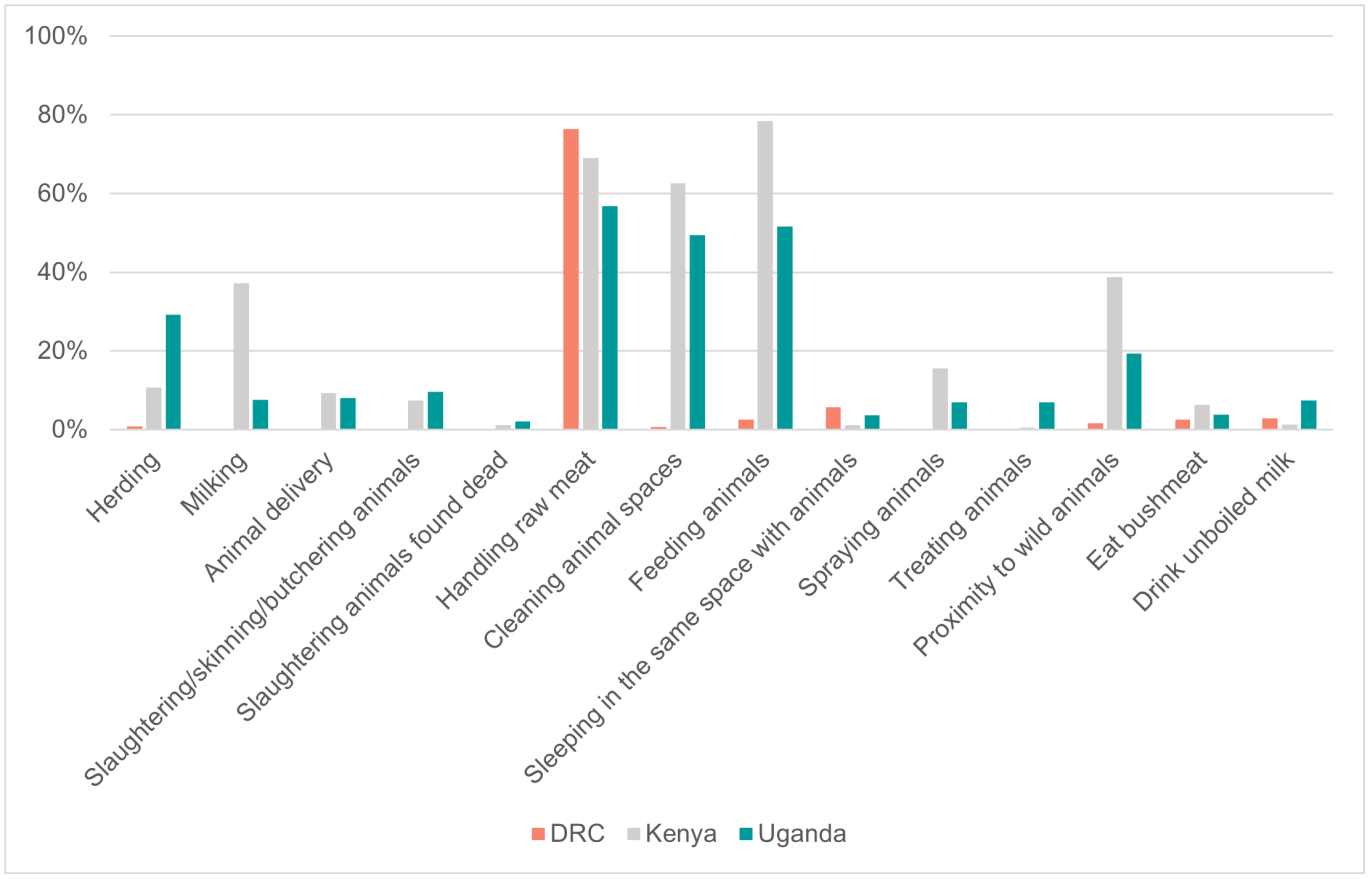
Human practices towards livestock reported by study participants, in the Democratic Republic of the Congo (DRC) (n = 1370), Kenya (n = 1468) and Uganda (n = 1968), 2021-2024.

The type of contact with animals, different livestock species and attitudes and practices towards livestock reported in the ECA cohorts are shown in Figs 2 and 3.

Overall, occupational activities involving contact with animals occurred less frequently among participants from the DRC than those from the other two countries. Nevertheless, over half of the overall population (63.4%) reported having animals within their household, which was again more prevalent in Kenya (84.7%) and Uganda (76.8%) than in the DRC (21.2%). Similarly, almost the entire study population (90.1%) reported having close contact with animals, predominantly in Kenya (96.2%) and Uganda (90.6%) where the exposures to livestock species, either as close contact or as animal ownership, was mainly with cattle (>60.0%), goats (>50.0%) and sheep (<10.0%) in Kenya, and goats (>50.0%), cattle (≃ 40.0%) and sheep (>20.0%) in Uganda. In the DRC, although animal contact was reported in 82.8% of cases, contact with poultry prevailed (>50.0%), while exposure to goats (>20.0%), cattle and sheep (<10.0%) were less frequent.

Consistent with this finding, the cohorts from Kenya and Uganda similarly reported a larger involvement in activities such as *feeding animals* (78.4% and 51.6%, respectively) and *cleaning animal spaces* (62.7% and 49.5%, respectively). While *milking* activities were predominantly carried out in Kenya (37.3%), participants in Uganda were more often engaged in *herding* (29.2%) and practicing activities such as *drinking unboiled milk* (7.5%). In the DRC, all these activities were all under-represented (≤5.0%), except for *handling raw meat during meal preparation*, which was reported by over three quarters of the participants (76.3%). About one-fifth of participants reported *proximity to wild animals* (20.2%), mainly in Kenya (38.8%) where also *eating wild animals* was most recorded (6.3%). In line with earlier findings, reports of herd abortions were minimal in the DRC (0.2%). Conversely, Kenya and Uganda reported a higher occurrence of both herd abortions and livestock mortality (respectively, 8.3% and 10.8% in Kenya and 9.0%and 15.7% in Uganda). Far less well known were the cases of unexplained death in wild animals, barely reported in Kenya (0.9%) and Uganda (0.8%) and completely absent in the DRC cohort.

### Mosquito vector exposure

**Fig 4:**
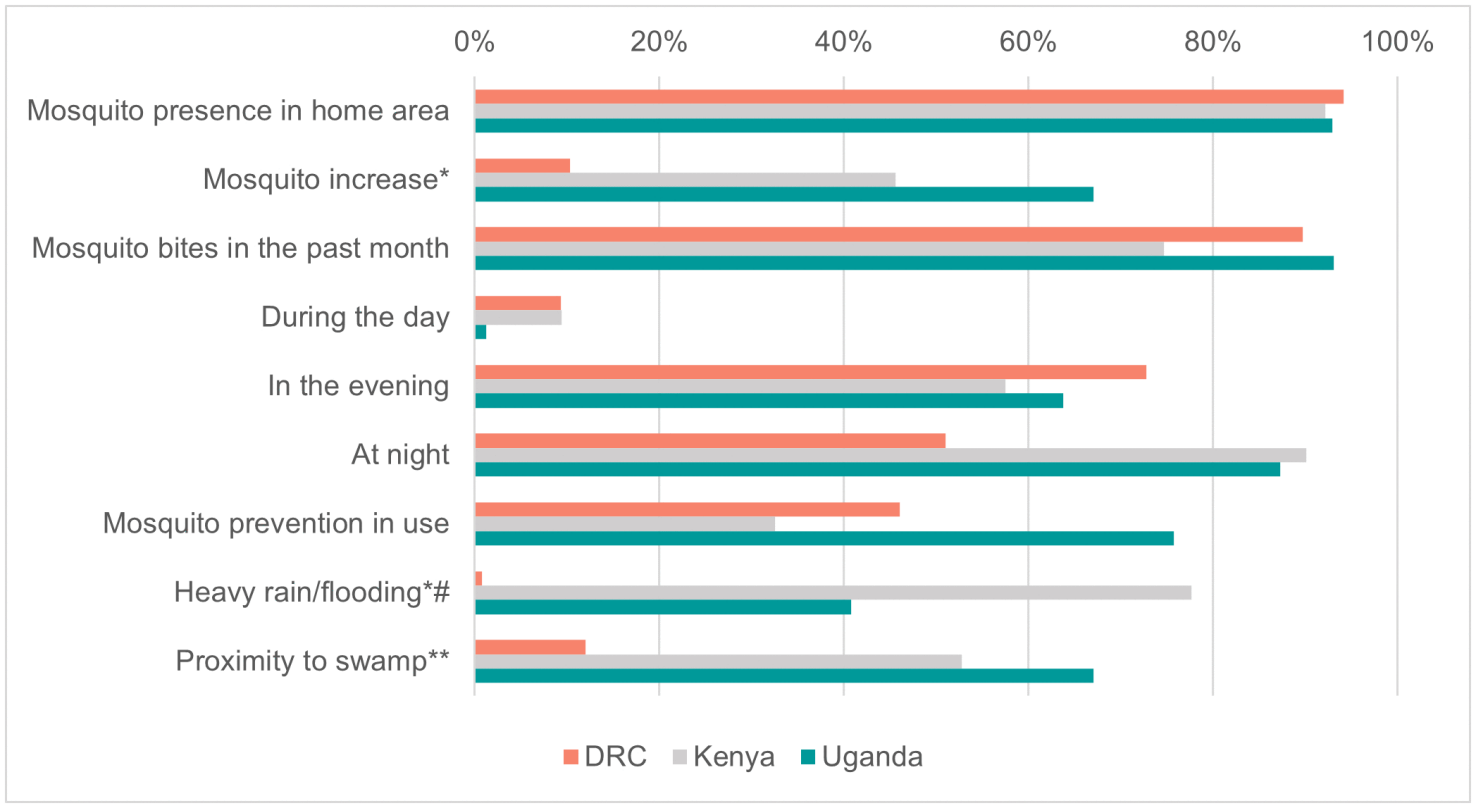
Mosquito exposure and living environments reported by study participants, in the Democratic republic of the Congo (DRC) (n = 1370), Kenya (n = 1468) and Uganda (n = 1968), 2021-2024. *around home area in the past two months; **within 20 km radius of home area, # reply available for population subset (n = 3,194).

The possible exposure to mosquito vectors and related living environment were also investigated (Fig 4). As expected, almost the entire population reported the presence of mosquitoes in their area of residence (93.0%) and having been bitten (86.5%) in the past month, predominantly during the night (77.8%) and evening (64.4%) hours. In the DRC only, evening hours (72.8%) were reported to be the time of higher mosquito nuisance than nighttime (51.0%). Nevertheless, only slightly more than half of the population (54.1%) reported using at least one mosquito protection measures, predominantly in Uganda (75.8%) and in the DRC (46.1%), compared to Kenya (32.5%). Environmental conditions with possible impact on the presence of mosquitoes, such as the presence of swamp, and recent episodes of heavy rain/flooding, were mentioned by over 40.0% of the participants enrolled in Uganda (67.1% and 40.7%, respectively) and Kenya (52.8% and 77.7%, respectively), lower in DRC (12.0% and 0.7%, respectively).

### Factors associated with RVF virus exposure in the overall population and by country

Factors associated with RVF positivity (being defined as having at least one RVF specific test being positive) were assessed for the overall population and by country.

Considering the overall study population of the three cohorts together, several factors emerged to be associated with RVF positivity during bivariate analysis (S1 Table). In the multivariate analysis, residence in Uganda, age range above 20 years old, male gender, and occupational activities were independently associated with exposure to RVF (Table 2). In particular, participants in the Uganda cohort had about a 6-fold increased likelihood of having been exposed to RVF and older age (> 40 years) was most strongly associated with evidence of exposure during life (Table 2). S*praying animals* and working as a butcher and/or in slaughterhouse were associated with RVF positivity during the pan-site analyses; however, *treating animals* was negatively associated (Table 2). Among the different livestock species investigated in our study, only contact with sheep was found to be significantly associated (aOR 1.59, 95% CI 1.06-2.37, p 0.023), unlike contact with cattle/goats. No environmental nor vector-related conditions showed significant association with RVF positivity.

**Table 2:** Factors associated with Rift Valley fever positivity, bivariate and multivariate analysis of the overall study population in the DRC, Kenya and Uganda, (n = 4806), 2021-2024.

Variable	Overall	Negative	Positive	cOR (95% CI)	p-value	aOR (95% CI)	p-value
Country DRC	1370 (28.5)	1,351 (29.7)	19 (7.5)			ref	*ref*
Kenya	1468 (30.5)	1,439 (31.6)	29 (11.5)	1.43 (0.81-2.61)	0.2	0.79 (0.42-1.51)	0.5
Uganda	1968 (41.0)	1763 (38.7)	205 (81.0)	8.27 (5.28-13.74)	<0.001	**5.94 (3.56-10.4)**	**<0.001**
Age-group, years 10–20	925 (19.2)	896 (19.7)	29 (11.5)			ref	*ref*
21-40	2,494 (51.9)	2,367 (52.0)	127 (50.2)	1.66 (1.12-2.54)	0.016	**1.73 (1.14-2.70)**	**0.012**
Above 40	1,387 (28.9)	1,290 (28.3)	97 (38.3)	2.32 (1.54-3.61)	<0.001	**2.60 (1.67-4.16)**	**<0.001**
Gender Female	2,763 (57.5)	2,649 (58.2)	114 (45.1)			ref	*ref*
Male	2,043 (42.5)	1,904 (41.8)	139 (54.9)	1.70 (1.32-2.19)	<0.001	**1.60 (1.22-2.10)**	**<0.001**
Crop Farmer No	3,173 (66.0)	3,075 (67.5)	98 (38.7)			ref	*ref*
Yes	1,633 (34.0)	1,478 (32.5)	155 (61.3)	3.29 (2.54-4.28)	<0.001	**1.39 (1.02-1.89)**	**0.037**
Butcher No	4,773 (99.3)	4,526 (99.4)	247 (97.6)			ref	*ref*
Yes	33 (0.7)	27 (0.6)	6 (2.4)	4.07 (1.51-9.31)	0.002	**3.10 (1.07-7.81)**	**0.024**
Spraying animals No	4,439 (92.4)	4,215 (92.6)	224 (88.5)			ref	*ref*
Yes	367 (7.6)	338 (7.4)	29 (11.5)	1.61 (1.06-2.38)	0.020	**2.44 (1.34-4.35)**	**0.003**
Treating animals No	4,659 (96.9)	4,420 (97.1)	239 (94.5)			ref	*ref*
Yes	147 (3.06)	133 (2.9)	14 (5.5)	1.95 (1.06-3.31)	0.021	**0.40 (0.18-0.86)**	**0.021**
Keeping sheep No	4,113 (85.6)	3,929 (86.3)	184 (72.7)			ref	*ref*
Yes	693 (14.4)	624 (13.7)	69 (27.3)	2.36 (1.76-3.14)	<0.001	0.71 (0.48-1.05)	0.092
Contact with sheep No	4,187 (87.1)	4.001 (87.9)	186 (73.5)			ref	*ref*
Yes	619 (12.9)	552 (12.1)	67 (26.5)	2.61 (1.94-3.48)	<0.001	**1.59 (1.06-2.37)**	**0.023**
Contact with goat No	2,465 (51.3)	2,372 (52.1)	93 (36.8)			ref	*ref*
Yes	2,341 (48.7)	2,181 (47.9)	160 (63.2)	1.87 (1.44-2.44)	<0.001	1.31 (0.99-1.75)	0.064

aOR, adjusted Odds Ratio; CI, Confidence Interval; cOR, crude Odds Ratio; DRC, Democratic Republic of the Congo; ref, reference

The same variables were also assessed for possible association with RVF exposure by country (Table 3). The complete bivariate analyses by country are presented in the supplementary material (S2, S3, S4 Tables). For the DRC cohort, the age group, being an animal farmer and *herding*, showed a trend approaching a significant association with RVF positivity (p-value <0.1) (S2 Table), but none achieved statistical significance (p > 0.05) at multivariate analysis (Table 3). Apart from one positive subject in the 10–20 years old age group, the other 18 positive individuals were equally distributed in the two older age-groups. Positive individuals were mainly women (63.2%) with a minimal gap between subjects who reported high or low schooling (42.1% and 57.9% respectively, p-value 0.4). Apart from one subject (5.3%) who reported having travelled in the last two months within the same district, the remaining 18 subjects (94.7%) did not report any recent trips inside or outside the DRC. In the cohort from the DRC, none of the occupational activities or activities involving contact with livestock showed a significant association with exposure to RVF virus, nor did any environmental factor or exposure to the mosquito vector.

**Table 3:** Factors associated with Rift Valley fever positivity, in the DRC (n = 1370), Kenya (n = 1468) and Uganda (n = 1968), 2021-2024.

	DRC *N tot 1,370 – N pos 19*				KENYA *N tot 1,468 – N pos 29*				UGANDA *N tot 1,968 – N pos 205*			
Variable	Negative	Positive	aOR (95% CI)	p-value	Negative	Positive	aOR (95% CI)	p-value	Negative	Positive	aOR (95% CI)	p-value
Age-group, 10–20 years	242 (17.9)	1 (5.3)	*ref*		253 (17.6)	2 (6.9)	*ref*		401 (22.7)	26 (12.7)	*ref*	
21-40 years	766 (56.7)	9 (47.4)	2.78 (0.51-51.8)	0.3	672 (46.7)	7 (24.1)	1.20 (0.27-8.26)	0.8	929 (52.7)	111(54.1)	**1.95 (1.27-3.11)**	**0.003**
Above 40 years	343 (25.4)	9 (47.4)	6.36 (1.18-118)	0.081	514 (35.7)	20 (69.0)	4.32 (1.10-28.80)	0.064	433 (24.6)	68 (33.2)	**2.54 (1.60-4.14)**	**<0.001**
Male gender *(ref = female)*	424 (31.4)	7 (36.8)	–	–	710 (49.3)	24 (82.8)	**7.12 (2.65-22.8)**	**<0.001**	770 (43.7)	108 (52.7)	**1.44 (1.07-1.93)**	**0.015**
HCW *(ref = No)*	94 (7.0)	3 (15.8)	–	–	16 (1.1)	1 (3.5)	–	–	66 (3.7)	2 (1.0)	**0.24 (0.04-0.78)**	**0.049**
Animal farmer *(ref = No)*	10 (0.7)	1 (5.3)	5.97 (0.30-37.6)	0.11	343 (23.8)	12 (41.4)	–	–	263 (14.9)	39 (19.0)	**–**	**–**
Crop Farmer *(ref = No)*	36 (2.7)	1 (5.3)	–	–	386 (26.8)	14 (48.3)	**2.40 (1.01-5.68)**	**0.046**	1,056 (59.9)	140 (68.3)	–	**–**
Butcher *(ref = No)*	3 (0.22)	0 (0.0)	–	–	13 (0.9)	2 (6.9)	4.72 (0.64-22.4)	0.074	11 (0.6)	4 (1.9)	–	**–**
Herding *(ref = No)*	11 (0.8)	1 (5.3)	5.60 (0.28-35.3)	0.13	150 (10.4)	7 (24.1)	**–**	**–**	516 (29.3)	59 (28.8)	–	**–**
Milking *(ref = No)*	0 (0.0)	0 (0.0)	–	–	526 (36.5)	21 (72.4)	**3.20 (1.38-8.04)**	**0.009**	131 (7.4)	18 (8.8)	–	–
Abortions in herds *(ref = No)*	3 (0.2)	0 (0.0)	–	–	114 (7.9)	8 (27.6)	**2.95 (1.13-7.15)**	**0.020**	160 (9.1)	18 (8.8)	–	–
Mosquito prevent *(ref = No)*	621 (46.0)	10 (52.6)	–	–	472 (32.8)	5 (17.2)	0.37 (0.12-0.94)	0.054	1,337 (75.8)	154 (75.1)	–	–
Goat contact *(ref = No)*	329 (24.3)	6 (31.6)	–	–	858 (59.6)	59.6 (75.9)	–	–	994 (56.4)	132 (64.4)	**1.39 (1.03-1.89)**	**0.034**

aOR, adjusted odds ratio; CI, Confidence Interval; DRC, Democratic Republic of the Congo; HCW, Healthcare worker; N tot, total number; N pos, number of positive; prevent, prevention; ref, reference

The bivariate analyses in the Kenyan and Ugandan cohorts (S3, S4 Tables) on the other hand, revealed a series of significant factors, albeit different in the two contexts, and which were further explored via country-specific multivariate analyses (Table 3). After adjusting for other variables, in the multivariate model for the cohort from Kenya, the over-40-year-old age group showed a potentially positive association with exposure to RVF virus, although not statistically significant (p 0.064) (Table 3). Male gender, being a crop farmer, milking activity and being aware of cases of abortion in livestock herds were associated with RVF positivity (Table 3). Borderline significant was the negative association with the use of at least one means of prevention against mosquito bites (p 0.054) (Table 3). The multivariate logistic regression for the Ugandan cohort indicated increased odds of RVF positivity with the 21–40 and over 40 year age groups and male gender (Table 3). Among occupational activities, being a health care worker was negatively associated, while contact with goats was positively associated with RVF positivity (Table 3).

## Discussion

The overall RVF positivity in our study population was 5.3%, with country-specific rates of 1.4% in DRC, 2.0% in Kenya and 10.4% in Uganda. Variations in RVF positivity across countries (p < 0.001) may reflect differences in patient characteristics, livestock contact, and exposure to infected vectors. In Kenya from 2008 to 2022, 41 RVF disease events were recorded as small clusters concentrated in the southwestern highlands [17], while Uganda’s high prevalence was consistent with the ongoing outbreak in 2021 that extended to our study area [32]. A review, based on ProMed data documented 67 RVF outbreaks between 2010 and 2024 in Uganda, Rwanda, Kenya, Tanzania, Burundi and South Sudan [8], but there were no reports of RVF outbreaks from the DRC. Apart from an article published in 1999, identifying RVF virus IgG antibodies in two Kisangani residents, in the central-eastern part of the DRC, to the best of our knowledge no other reports exist [33] and no RVF virus-related hemorrhagic fever events have been documented in humans to date. While severe bleeding cases would likely trigger investigation in the DRC, and more in particular in the North Kivu region, given the region’s vigilance for Ebola, such severe presentations do not seem to occur. Nonetheless, RVF virus circulation in animals in the DRC has been detected, both in cattle [34] and in small ruminants [18]. In particular, in the North Kivu, Goma region, a 2013 study reported 12.7% RVF IgG prevalence and 2.0% RVF IgM prevalence in cattle, indicating the presence of a potential recent/silent circulation [19]. Taken together, these data suggest a potentially inadequate RVF surveillance system with possible underdiagnosis and/or underreporting, especially for milder cases that may go undetected. A shift in the RVF epidemiology has been recently suggested, and is presented as a dynamic spectrum, ranging from epidemic-prone regions with large outbreaks interspersed with interepidemic periods, to hyperendemic regions characterized by frequent infections and small or absent outbreaks [35]. Based on our current knowledge of RVF in the DRC, it remains currently difficult to determine where the DRC falls on this spectrum and challenging to position it at a single point, given the country’s large size and diverse ecological zones. Furthermore, the coexistence of multiple health emergencies, and the complex humanitarian crisis, particularly in the eastern provinces, further complicate the epidemiological landscape and surveillance efforts in the country. Nevertheless, our findings in humans (1.4%), though low and not providing evidence of ongoing transmission, warrant attention due to potential effects of climate change on RVF dynamics. Moreover, consideration should be given to our study population that was not limited to the RVF high-risk categories commonly targeted in other studies, namely butchers, slaughterhouse workers or animal farmers [36–38], but rather on a cohort of patients with acute fever, capturing a broader vulnerable population, including subjects aged 10 and over, being the age-level at which children become active in the livestock sector in rural areas.

Cross-country comparisons revealed differences in livestock contact. Overall, participants from the DRC reported significantly less occupational contact with livestock, whether participants kept animals in their household or were involved in activities with livestock, compared to Kenya and Uganda. Although over 80% of the Congolese study population reported contact with animals, this mainly referred to the handling of raw meat during meal preparation, which nevertheless carries a potential risk of RVF transmission [27]. Furthermore, contact with animals in the DRC cohort was mainly reported with poultry, whereas cattle, goats and sheep, typically affected by RVF, were more commonly reported in the Kenyan and Ugandan cohorts. Given these findings, targeted studies in DRC high-risk populations, such as butchers, slaughterhouse or farm workers, are warranted to confirm exposure patterns and risk factors.

Although a similar seropositivity was evidenced in Kenya and in the DRC, it was possible to find statistical significant associations with different factors in Kenya, probably due to a higher sample size, which was not the case for the DRC. Hence a larger study in the DRC is needed, as suggested in high risk populations, to confirm or not the association with risk factors as observed in the other two countries.

Several differences among our three cohorts were observed. In the DRC, higher RVF positivity was identified among females (63.2%), although not statistically significant, contrasting Kenya and Uganda, where males were significantly more exposed. Men are, indeed, typically considered to be more involved in livestock management and slaughter: a meta-analysis of studies conducted between 1999 and 2021, estimated that males had 1.41 times higher odds of exposure than females (95% CI 1.24, 1.60, p < 0.001) [39]. However, gender-specific behaviors and roles vary by context, altering this pairing and emphasizing the need for culturally tailored prevention strategies. [36]. Age-related trends also differed (S1 Fig). In the DRC, apart from one subject in the 10–20 age group, RVF positivity was equally distributed between the 21–40 and over 40 age groups. In Kenya, older adults (>40 years) had higher exposure, likely due to past epidemics, while in Uganda, the 21–40 age group showed greater exposure, possibly reflecting ongoing transmission.

The analysis highlighted distinct exposure patterns in Kenya and Uganda. For the Kenyan cohort, similar to what was identified in a previous paper by Situma et al., which included the same population of febrile subjects combined with farmers from the same area [20], milking and awareness of abortion cases in herds were found to be significantly associated with exposure to RVF during life. A previous study conducted in Kenya highlighted the potential exposure to RVF through raw milk which could hence be a risk factor for the urban population, without direct contact with livestock [40]. Similarly, being aware of abortions in herds as a risk factor might provide an opportunity for community-based surveillance for early detection of RVF outbreaks [12,41]. In the Ugandan cohort, being a health care worker had a strong negative association, which may be consistent with both a decreased likelihood of direct exposure to livestock among health care workers, as well as perhaps an increased tendency to live in environments not associated with farming and husbandry activities, as well as the absence to date of reports of human-to-human transmission of RVF. In contrast, health care-associated transmission of Ebola Sudan virus of the 2022 outbreak in Uganda, during our study period, was reported to be important [42]. Finally, only in the Ugandan cohort, contact with goats was associated with exposure to RVF virus. Although sheep are often considered the species most susceptible to RVF, a study conducted in Kenya also found that being associated with an IgG-positive goat herd increased the odds of RVF seropositivity by 3.8-fold (95% CI 1.17–12.3) and that infections in goats could also provide information on the likely occurrence of the disease in human populations [43]. Expanding this analysis to our study area in the DRC would certainly be beneficial given that nearly a quarter of our Congolese cohort reported contact with goats, far greater than that reported with cattle and sheep. A previous study in seven provinces of the DRC identified a seroprevalence of 23.81% (95% CI 12.03–41.76) in goats, but North Kivu province, the one of our study, was not included [18].

In the multivariate analysis of all three study cohorts, two activities associated with RVF virus exposure were subject to non-definitive interpretation, with *treatment animals* as protective and, conversely, *spraying* animals as risky. It is possible that spraying was more common in areas with a higher density of mosquitoes and therefore a higher risk of transmission. Likewise, it is possible that treating animals included measures to prevent exposure to vectors and vaccinations, which would explain a reduction in the odds. Further studies need to be done to confirm this hypothesis, and also the importance of activities other than slaughtering or handling abortions as demonstrated by a previous report of a 25-year-old cattle herder in Uganda who died from RVF and who was mainly involved in cattle grazing, milking, and spraying for control of ticks and other pests [44].

Although participants reported a wide presence of mosquitoes in the three study areas and proximity to swampy areas, that could favor mosquito exposure, none of these were significantly associated with RVF positivity. This underscores the need for integrated One Health approaches incorporating entomological surveillance [45], to better understand local transmission dynamics, especially under changing climatic conditions.

A key strength of this study was the multi-country design within the same region, with coordination between the different study teams, and using standardized protocols for surveys and diagnostic testing to minimize possible risk of bias. Nevertheless, we cannot exclude the possibility that both recall bias and social desirability bias happened during the survey. Another limitation related to the sample size of the DRC cohort, which was interrupted 4 months before the expected end-date due to financial reasons, resulted in a limited decrease of the statistical power for this country, counterbalanced by the higher final sample (above the calculated sample size) in the other countries. Finally, we cannot exclude the potential underestimation of our seroprevalence findings based on the exclusion of children under 10 years of age from our study. Although children are less likely than adults to be exposed to high-risk activities associated with RVF virus transmission, the risk cannot be completely ruled out [46].

## Conclusions

In conclusion, RVF transmission in ECA also concerns the DRC, where no human cases have previously been documented. The 1.4% among febrile patients in the DRC cohort, indicates low but existing exposure. Differences in prevalence between DRC, Kenya, and Uganda might reflect varying levels of animal contact and environmental risk or a true difference in RVF prevalence. Therefore, the risk factors identified in the neighboring countries, Kenya and Uganda, need to be further studied in the context of the DRC, specifically targeting the most at-risk human and animal populations. Incorporating One Health approaches—linking human, animal, and vector data—will be crucial to elucidate RVF ecology in the region. Understanding how climate change, human and animal mobility, and ecological shifts affect RVF transmission can inform forecasting models and preventive strategies to mitigate future outbreaks in East and Central Africa.

## Supporting information

S1 File Strobe checklist. (PDF)

S1 Table Bivariate analysis by Rift Valley fever positivity for the overall study population in the Democratic Republic of the Congo, Kenya and Uganda, (n = 4806), 2021–2024. Legend: CI, confidence Interval; cOR, crude Odds Ratio; DRC, Democratic Republic of the Congo. *In the last 2 months, **within 20 km radius of home area.
(PDF)

S2 Table Bivariate analysis by Rift Valley fever positivity for the cohort from the Democratic Republic of the Congo (DRC). Legend: CI, confidence Interval; cOR, crude Odds Ratio; DRC, Democratic Republic of the Congo. *In the last 2 months, **within 20 km radius of home area.
(PDF)

S3 Table Bivariate analysis by Rift Valley fever positivity for the cohort from Kenya. Legend: CI, confidence Interval; cOR, crude Odds Ratio. *In the last 2 months, **within 20 km radius of home area.
(PDF)

S4 Table Bivariate analysis by Rift Valley fever positivity for the cohort from Uganda. Legend: CI, confidence Interval; cOR, crude Odds Ratio. *In the last 2 months, **within 20 km radius of home area.
(PDF)

S1 Fig Age Distribution of Rift Valley fever positivity by Country, in the Democratic Republic of the Congo (DRC), Kenya and Uganda, (n = 4806), 2021–2024. (TIF)
