# Supplementary material for: Understanding the Rift Valley fever exposure risk: A comparative perspective from a multi-country study in East and Central Africa, 2021-24
Source: PLoS Negl Trop Dis. 2026 Mar 10;20(3):e0014082. doi: 10.1371/journal.pntd.0014082 (PMC12987580; doi:10.1371/journal.pntd.0014082)
Supplement: S1 Table — Legend: CI, confidence Interval; cOR, crude Odds Ratio; DRC, Democratic Republic of the Congo. *In the last 2 months, **within 20 km radius of home area. (PDF) [file pntd.0014082.s002.pdf]

S1 Table. Bivariate analysis by Rift Valley fever positivity for the overall study population in the Democratic Republic of the Congo, Kenya and Uganda, (n=4806), 2021-2024.

Variable		Overall	Negative	Positive	cOR (95% CI)	p-value
Country	DRC	1370 (28.5)	1,351 (29.7)	19 (7.5)		
	Kenya	1468 (30.5)	1,439 (31.6)	29 (11.5)	1.43 (0.81-2.61)	0.2
	Uganda	1968 (41.0)	1763 (38.7)	205 (81.0)	8.27 (5.28-13.74)	<0.001
Age-group	10-20 years old	925 (19.2)	896 (19.7)	29 (11.5)		
	21-40 years old	2,494 (51.9)	2,367 (52.0)	127 (50.2)	1.66 (1.12-2.54)	0.016
	Above 40 years old	1,387 (28.9)	1,290 (28.3)	97 (38.3)	2.32 (1.54-3.61)	<0.001
Gender	Female	2,763 (57.5)	2,649 (58.2)	114 (45.1)		
	Male	2,043 (42.5)	1,904 (41.8)	139 (54.9)	1.70 (1.32-2.19)	<0.001
Education	High Schooling	1,761 (36.6)	1,701 (37.4)	60 (23.7)		
	Low Schooling	3,045 (63.4)	2,852 (62.6)	193 (76.3)	1.92 (1.44-2.60)	<0.001
Healthcare worker	No	4,624 (96.2)	4,377 (96.1)	247 (97.6)		
	Yes	182 (3.8)	176 (3.9)	6 (2.4)	0.60 (0.24-1.26)	0.2
Farmer (animal husbandry)	No	4,138 (86.1)	3,937 (86.5)	201 (79.5)		
	Yes	668 (13.9)	616 (13.5)	52 (20.5)	1.65 (1.19-2.25)	0.002
Farmer (crop cultivation)	No	3173 (66.0)	3,075 (67.5)	98 (38.7)		
	Yes	1,633 (34.0)	1,478 (32.5)	155 (61.3)	3.29 (2.54-4.28)	<0.001
Butcher	No	4,773 (99.3)	4,526 (99.4)	247 (97.6)		
	Yes	33 (0.7)	27 (0.6)	6 (2.4)	4.07 (1.51-9.31)	0.002
Similar disease in family*	No	4,229 (88.0)	4021 (88.3)	208 (82.2)		
	Yes	577 (12.0)	532 (11.7)	45 (17.8)	1.63 (1.16-2.26)	0.004
Similar disease community*	No	4,444 (92.5)	4,215 (92.6)	229 (90.5)		
	Yes	362 (7.5)	338 (7.4)	24 (9.5)	1.31 (0.83-1.98)	0.2
Keeping cattle	No	3,039 (63.2)	2,899 (63.7%)	140 (55.3)		
	Yes	1,767 (36.8)	1,654 (36.3%)	113 (44.7)	1.41 (1.09-1.82)	0.008
Keeping sheep	No	4,113 (85.6)	3,929 (86.3)	184 (72.7)		
	Yes	693 (14.4)	624 (13.7)	69 (27.3)	2.36 (1.76-3.14)	<0.001
Keeping goat	No	2,846 (59.2)	2,736 (60.1)	110 (43.5)		
	Yes	1,960 (40.8)	1,817 (39.9)	143 (56.5)	1.96 (1.52-2.53)	<0.001
Contact with cattle	No	2819 (58.7)	2,685 (59.0)	134 (53.0)		
	Yes	1,987 (41.3)	1868 (41.0)	119 (47.0)	1.28 (0.99-1.64)	0.059
Contact with sheep	No	4,187 (87.1)	4,001 (87.9)	186 (73.5)		
	Yes	619 (12.9)	552 (12.1)	67 (26.5)	2.61 (1.94-3.48)	<0.001
Contact with goat	No	2,465 (51.3)	2,372 (52.1)	93 (36.8)		
	Yes	2,341 (48.7)	2,181 (47.9)	160 (63.2)	1.87 (1.44-2.44)	<0.001
Herding animals	No	4,062 (84.5)	3,876 (85.1)	186 (73.5)		
	Yes	744 (115.5)	677 (14.9)	67 (26.5)	2.06 (1.53-2.74)	<0.001
Milking animals	No	4,110 (85.5)	3,896 (85.6)	214 (84.6)		
	Yes	696 (14.5)	657 (14.4)	39 (15.4)	1.08 (0.75-1.52)	0.7
Assisting animal birthing	No	4,511 (93.9)	4,288 (94.2)	223 (88.1)		
	Yes	295 (6.1)	265 (5.8)	30 (11.9)	2.18 (1.43-3.20)	<0.001
Slaughtering/skinning/	No	4,506 (93.8)	4281 (94.0)	225 (88.9)		
butchering animals	Yes	300 (6.2%)	272 (6.0)	28 (11.1)	1.96 (1.27-2.91)	0.003
Handling raw meat	No	1,628 (33.9)	1,519 (33.4)	109 (43.1)		
	Yes	3,178 (66.%)	3,034 (66.6)	144 (56.9)	0.66 (0.51-0.86)	0.002
Cleaning animal areas	No	2,903 (60.4)	2777 (61.0)	126 (49.8)		
	Yes	1,903 (39.6)	1,776 (39.0)	127 (50.2)	1.58 (1.22-2.03)	<0.001
Feeding animals	No	2,604 (54.2)	2,496 (54.8)	108 (42.7)		
	Yes	2202 (45.8)	2057 (45.2)	145 (57.3)	1.63 (1.27-2.11)	<0.001
Sleeping with animals	No	4638 (96.5)	4392 (96.5)	246 (97.2)		
	Yes	168 (3.5)	161 (6.5)	7 (2.8)	0.78 (0.33-1.55)	0.5
Spraying animals	No	4,439 (92.4)	4,215 (92.6)	224 (88.5)		
	Yes	367 (7.6)	338 (7.4)	29 (11.5)	1.61 (1.06-2.38)	0.020
Treating animals	No	4,659 (96.9)	4,420 (97.1)	239 (94.5)		
	Yes	147 (3.06)	133 (2.9)	14 (5.5)	1.95 (1.06-3.31)	0.021
Proximity to wild animals	No	3,836 (79.8)	3,641 (80.0)	195 (77.1)		
	Yes	970 (20.2)	912 (20.0)	58 (22.9)	1.19 (0.87-1.59)	0.3
Unusual illness humans	No	4,535 (94.4)	4,308 (94.6)	227 (89.7)		
	Yes	271 (5.6)	245 (5.4)	26 (10.3)	2.01 (1.29-3.03)	0.003
Unexplained human deaths	No	4,590 (95.5)	4,364 (95.9)	226 (89.3)		
	Yes	216 (4.5)	189 (4.1)	27 (10.7)	2.76 (1.77-4.15)	<0.001

Abortion in herds	No	4,503 (93.7)	4,276 (93.9)	227 (89.7)		
	Yes	303 (6.3)	277 (6.1)	26 (10.3)	1.77 (1.13-2.65)	0.008
Unexplained deaths in herds	No	4,337 (90.2)	4,128 (90.7)	209 (82.6)		
	Yes	469 (9.8)	425 (9.3)	44 (17.4)	2.04 (1.44-2.85)	<0.001
Unexplained deaths in wild animal	No	4,776 (99.4)	4,527 (99.4)	249 (98.4)		
	Yes	30 (0.6)	26 (0.6)	4 (1.6)	2.80 (0.82-7.25)	0.057
Slaughtering dead animals	No	4,748 (98.8)	4,501 (98.9)	247 (98.6)		
	Yes	58 (1.2)	52 (1.1)	6 (2.4)	2.10 (0.80-4.57)	0.088
Eat bushmeat	No	4,604 (95.8)	4.366 (95.9)	238 (94.1)		
	Yes	202 (4.2)	187 (4.1)	15 (5.9)	1.47 (0.82-2.45)	0.2
Drink raw milk	No	4,600 (95.7)	4,365 (95.9)	235 (92.9)		
	Yes	206 (4.3)	188 (4.1)	18 (7.1)	1.78 (1.04-2.86)	0.024
Mosquito bites	No	649 (13.5)	625 (13.7)	24 (9.5)		
	Yes	4,157 (86.5)	3,928 (86.3)	229 (90.5)	1.52 (1.01-2.39)	0.056
Mosquito prevention	No	2,207 (45.9)	2,123 (46.6)	84 (33.2)		
	Yes	2,599 (54.1)	2,430 (53.4)	169 (66.8)	1.76 (1.35-2.31)	<0.001
Mosquito presence in home area	No	334 (6.9)	314 (6.9)	20 (7.9)		
	Yes	4,472 (93.1)	4,239 (93.1)	233 (92.1)	0.86 (0.55-1.42)	0.5
Mosquito increase in home area*	No	2,677 (55.7)	2,578 (56.6)	99 (39.1)		
	Yes	2,129 (44.3)	1,975 (43.4)	154 (60.9)	2.03 (1.57-2.63)	<0.001
Proximity swamp**	No	2,547 (53.0)	2,458 (54.0)	89 (35.2)		
	Yes	2,259 (47.0)	2,095 (46.0)	164 (64.8)	2.16 (1.66-2.82)	<0.001

Legend: CI, confidence Interval; cOR, crude Odds Ratio; DRC, Democratic Republic of the Congo.

*In the last 2 months, **within 20 km radius of home area
