# Supplementary material for: Understanding the Rift Valley fever exposure risk: A comparative perspective from a multi-country study in East and Central Africa, 2021-24
Source: PLoS Negl Trop Dis. 2026 Mar 10;20(3):e0014082. doi: 10.1371/journal.pntd.0014082 (PMC12987580; doi:10.1371/journal.pntd.0014082)
Supplement: S2 Table — Legend: CI, confidence Interval; cOR, crude Odds Ratio; DRC, Democratic Republic of the Congo. *In the last 2 months, **within 20 km radius of home area. (PDF) [file pntd.0014082.s003.pdf]

S2 Table. Bivariate analysis by Rift Valley fever positivity for the cohort from the Democratic Republic of the Congo (DRC).

Variable		Overall	Negative	Positive	cOR (95% CI)	p-value
Age-group	10-20 years old	243 (17.7)	242 (17.9)	1 (5.3)		
	21-40 years old	775 (56.7)	766 (56.7)	9 (47.4)	2.84 (0.53-52.6)	0.3
	Above 40 years old	352 (25.7)	343 (25.4)	9 (47.4)	6.35 (1.18-117.49)	0.080
Gender	Female	939 (68.5)	927 (68.6)	12 (63.2)		
	Male	431 (31.5)	424 (31.4)	7 (36.8)	1.27 (0.47-3.19)	0.6
Education	High Schooling	702 (51.2)	694 (51.4)	8 (42.1)		
	Low Schooling	668 (48.8)	657 (48.6)	11 (57.9)	1.45 (0.58-3.77)	0.4
Healthcare worker	No	1,273 (92.9)	1,257 (93.0)	16 (84.2)		
	Yes	97 (7.1)	94 (7.0)	3 (15.8)	2.51 (0.58-7.69)	0.15
Farmer (animal husbandry)	No	1,359 (99.2)	1,341 (99.3)	18 (94.7)		
	Yes	11 (0.8)	10 (0.7)	1 (5.26)	7.45 (0.40-42.10)	0.062
Farmer (crop cultivation)	No	1,333 (97.3)	1315 (97.3)	18 (94.7)		
	Yes	37 (2.7)	36 (2.7)	1 (5.3)	2.03 (0.11-10.25)	0.5
Butcher	No	1,367 (99.8)	1,348 (99.8)	19 (100.0)		
	Yes	3 (0.2)	3 (0.22)	0 (0.0)	-	-
Similar disease in family*	No	1,293 (94.4)	1,275 (94.4)	18 (94.7)		
	Yes	77 (5.6)	76 (5.6)	1 (5.3)	0.93 (0.05-4.61)	0.9
Similar disease community*	No	1,349 (98.5)	1,330 (98.4)	19 (100.0)		
	Yes	21 (1.5)	21 (1.6)	0 (0.0)	-	-
Keeping cattle	No	1,368 (99.8)	1,349 (99.8)	19 (100.0)		
	Yes	2 (0.15)	2 (0.15)	0 (0.0)	-	-
Keeping sheep	No	1,363 (99.5)	1,344 (99.5)	19 (100.0)		
	Yes	7 (0.5)	7 (0.5)	0 (0.0)	-	-
Keeping goat	No	1,287 (93.9)	1268 (93.9)	19 (100.0)		
	Yes	83 (6.1)	83 (6.1)	0 (0.0)	-	-
Contact with cattle	No	1,293 (94.4)	1,275 (94.4)	18 (94.7)		
	Yes	77 (5.6)	76 (5.6)	1 (5.3)	0.93 (0.05-4.61)	0.9
Contact with sheep	No	1,334 (97.4)	1,316 (97.4)	18 (94.7)		
	Yes	36 (2.6)	35 (2.6)	1 (5.3)	2.09 (0.11-10.57)	0.5
Contact with goat	No	1,035 (75.5)	1,022 (75.6)	13 (68.4)		
	Yes	335 (24.5)	329 (24.3)	6 (31.6)	1.43 (0.50-3.66)	0.5
Herding animals	No	1,358 (99.1)	1,340 (99.2)	18 (94.7)		
	Yes	12 (0.88)	11 (0.8)	1 (5.3)	6.77 (0.36-37.70)	0.074
Milking animals	No	1,370 (100.0)	1,351 (100.0)	19 (100.0)		
	Yes	0 (0.0)	0 (0.0)	0 (0.0)	-	-
Assisting animal birthing	No	1,369 (99.9)	1,350 (99.9)	19 (100.0)		
	Yes	1 (0.1)	1 (0.1)	0 (0.0)	-	-
Slaughtering/skinning/ butchering animals	No<br>Yes	1,369 (99.9)<br>1 (0.1)	1,350 (99.9)<br>1 (0.1)	19 (100.0)<br>0 (0.0)	-	-
Handling raw meat	No	324 (23.6)	317 (23.5)	7 (36.8)		
	Yes	1,046 (76.4)	1,034 (76.5)	12 (63.2)	0.53 (0.21-1.42)	0.18
Cleaning animal areas	No	1,361 (99.4)	1,342 (99.3)	19 (100.0)		
	Yes	9 (0.6)	9 (0.7)	0 (0.0)	-	-
Feeding animals	No	1,334 (97.4)	1,316 (97.4)	18 (94.7)		
	Yes	36 (2.6)	35 (2.6)	1 (5.3)	2.09 (0.11-10.57)	0.5
Sleeping with animals	No	1,292 (94.3)	1,274 (94.3)	18 (94.7)		
	Yes	78 (5.7)	77 (5.7)	1 (5.3)	0.92 (0.05-4.54)	0.9
Spraying animals	No	1,369 (99.9)	1,350 (99.9)	19 (100.0)		
	Yes	1 (0.1)	1 (0.1)	0 (0.0)	-	-
Treating animals	No	1,367 (99.8)	1,348 (99.8)	19 (100.0)		
	Yes	3 (0.2)	3 (0.2)	0 (0.0)	-	-
Proximity to wild animals	No	1,348 (98.4)	1,329 (98.4)	19 (100.0)		
	Yes	22 (1.6)	22 (1.6)	0 (0.0)	-	-
Unusual illness in humans	No	1,366 (99.7)	1,347 (99.7)	19 (100.0)		
	Yes	4 (0.3)	4 (0.3)	0 (0.0)	-	-
Unexplained human deaths	No	1,367 (99.8)	1,348 (99.8)	19 (100.0)		
	Yes	3 (0.2)	3 (0.2)	0 (0.0)	-	-
Abortion in herds	No	1,367 (99.8)	1,348 (99.8)	19 (100.0)		
	Yes	3 (0.2)	3 (0.2)	0 (0.0)	-	-
Unexplained deaths in herds	No	1,368 (99.8)	1,349 (99.9)	19 (0.0)		
	Yes	2 (0.2)	2 (0.1)	0 (0.0)	-	-
Unexplained deaths in wild animal	No<br>Yes	1,370 (100.0)<br>0 (0.0)	1,351 (100.0)<br>0 (0.0)	19 (100.0)<br>0 (0.0)	-	-
Slaughtering dead animals	No	1,370 (100.0)	1,351 (100.0)	19 (100.0)	-	

	Yes	0 (0.0)	0 (0.0)	0 (0.0)	-	-
Eat bushmeat	No	1,334 (97.4)	1,316 (97.4)	18 (94.7)		
	Yes	36 (2.6)	35 (2.6)	1 (5.3)	2.09 (0.11-10.57)	0.5
Drink raw milk	No	1,331 (97.1)	1,312 (97.1)	19 (100.0)		
	Yes	39 (2.9)	39 (2.9)	0 (0.0)	-	-
Mosquito bites	No	141 (10.3)	140 (10.4)	1 (5.3)		
	Yes	1229 (89.7)	1,211 (89.6)	18 (94.7)	2.08 (0.42-37.58)	0.5
Mosquito prevention	No	739 (53.9)	730 (54.0)	9 (47.4)		
	Yes	631 (46.1)	621 (46.0)	10 (52.6)	1.31 (0.52-3.31)	0.6
Mosquito presence in home area	No	80 (5.8)	79 (5.9)	1 (5.3)		
	Yes	1290 (94.2)	1,272 (94.1)	18 (94.7)	1.12 (0.23-20.24)	0.9
Mosquito increase in home area*	No	1,229 (89.7)	1,211 (89.6)	18 (94.7)		
	Yes	141 (10.3)	140 (10.4)	1 (5.3)	0.48 (0.03-2.35)	0.5
Proximity swamp**	No	1,206 (88.0)	1188 (87.9)	18 (94.7)		
	Yes	164 (12.0)	163 (12.1)	1 (5.3)	0.40 (0.02-1.98)	0.4

Legend: CI, confidence Interval; cOR, crude Odds Ratio; DRC, Democratic Republic of the Congo.

*In the last 2 months, **within 20 km radius of home area
