# Supplementary material for: Understanding the Rift Valley fever exposure risk: A comparative perspective from a multi-country study in East and Central Africa, 2021-24
Source: PLoS Negl Trop Dis. 2026 Mar 10;20(3):e0014082. doi: 10.1371/journal.pntd.0014082 (PMC12987580; doi:10.1371/journal.pntd.0014082)
Supplement: S3 Table — Legend: CI, confidence Interval; cOR, crude Odds Ratio. *In the last 2 months, **within 20 km radius of home area. (PDF) [file pntd.0014082.s004.pdf]

S3 Table. Bivariate analysis by RVF positivity for the cohort from Kenya.

Variable		Overall	Negative	Positive	cOR (95% CI)	p-value
Age-group	10-20 years old	255 (17.4)	253 (17.6)	2 (6.9)		
	21-40 years old	679 (26.2)	672 (46.7)	7 (24.1)	1.32 (0.32-8.88)	0.7
	Above 40 years old	534 (36.4)	514 (35.7)	20 (69.0)	4.92 (1.42-30.98)	0.032
Gender	Female	734 (50.0)	729 (50.7)	5 (17.2)		
	Male	734 (50.0)	710 (49.3)	24 (82.8)	4.93 (2.03-14.70)	0.001
Education	High Schooling	555 (37.8)	548 (38.1)	7 (24.1)		
	Low Schooling	913 (62.2)	891 (61.9)	22 (75.9)	1.93 (0.86-4.92)	0.132
Healthcare worker	No	1,451 (98.8)	1,423 (98.9)	28 (96.5)		
	Yes	17 (1.2)	16 (1.1)	1 (3.5)	3.18 (0.17-16.42)	0.3
Farmer (animal husbandry)	No	1,113 (75.8)	1,096 (76.2)	17 (58.6)		
	Yes	355 (24.2)	343 (23.8)	12 (41.4)	2.26 (1.04-4.73)	0.033
Farmer (crop cultivation)	No	1,068 (72.7)	1,053 (73.2)	15 (51.7)		
	Yes	400 (27.2)	386 (26.8)	14 (48.3)	2.55 (1.20-5.35)	0.013
Butcher	No	1,453 (99.0)	1,426 (99.1)	27 (93.1)		
	Yes	15 (1.0)	13 (0.9)	2 (6.9)	8.13 (1.23-31.35)	0.008
Similar disease in family*	No	1,351 (92.0)	1,325 (92.1)	26 (89.7)		
	Yes	117 (8.0)	114 (7.9)	3 (10.3)	1.34 (0.32-3.88)	0.6
Similar disease in community*	No	1,395 (95.0)	1,367 (95.0)	28 (96.5)		
	Yes	73 (5.0)	72 (5.0)	1 (3.5)	0.68 (0.04-3.25)	0.7
Keeping cattle	No	559 (38.1)	548 (38.1)	11 (37.9)		
	Yes	909 (61.9)	891 (61.9)	18 (62.1)	1.01 (0.48-2.21)	1.0
Keeping sheep	No	1,389 (94.6)	1,361 (94.6)	28 (96.5)		
	Yes	79 (5.4)	78 (5.4)	1 (3,5)	0.62 (0.03-2.98)	0.6
Keeping goat	No	717 (48.8)	705 (49.0)	12 (41.4)		
	Yes	751 (51.2)	734 (51.0)	17 (58.6)	1.36 (0.65-2.94)	0.4
Contact with cattle	No	342 (23.3)	340 (23.6)	2 (6.9)		
	Yes	1,126 (76.7)	1,099 (76.4)	27 (93.1)	4.18 (1.24-25.98)	0.052
Contact with sheep	No	1,351 92.0)	1,327 (92.2)	24 (82.8)		
	Yes	117 (8.0)	112 (7.8)	5 (17.2)	2.47 (0.82-6.09)	0.071
Contact with goat	No	588 (40.1)	581 (40.4)	7 (24.1)		
	Yes	880 (59.9)	858 (59.6)	22 (75.9)	2.13 (0.95-5.41)	0.084
Herding animals	No	1,311 (89.3)	1,289 (89.6)	22 (15.9)		
	Yes	157 (10.7)	150 (10.4)	7 (24.1)	2.73 (1.07-6.21)	0.023
Milking animals	No	921 (62.7)	913 (63.5)	8 (27.6)		
	Yes	547 (37.3)	526 (36.5)	21 (72.4)	4.56 (2.08-11.02)	<0.001
Assisting animal birthing	No	1,331 (90.7)	1,309 (91.0)	22 (75.9)		
	Yes	137 (9.3)	130 (9.0)	7 (24.1)	3.20 (1.25-7.29)	0.009
Slaughtering/skinning/	No	1,358 (92.5)	1,336 (92.8)	22 (75.9)		
butchering animals	Yes	110 (7.5)	103 (7.2)	7 (24.1)	4.13 (1.60-9.44)	0.001
Handling raw meat	No	455 (31.0)	448 (31.1)	7 (24.1)		
	Yes	1,013 (69.0)	991 968.9)	22 (75.9)	1.42 (0.63-3.62)	0.4
Cleaning animal areas	No	548 (37.3)	540 (37.5)	8 (27.6)		
	Yes	920 (62.7)	899 (62.5)	21 (72.4)	1.58 (0.72-3.81)	0.3
Feeding animals	No	317 (21.6)	313 (21.7)	4 (13.8)		
	Yes	1,151 (78.4)	1,126 (78.3)	25 (86.2)	1.74 (0.67-5.93)	0.3
Sleeping with animals	No	1,450 (98.8)	1,421 (98.7)	29 (100.0)		
	Yes	18 (1.2)	18 (1.3)	0 (0.0)	-	-
Spraying animals	No	1,239 (84.4)	1,223 (85.0)	16 (55.2)		
	Yes	229 (15.6)	216 (15.0)	13 (44.8)	4.60 (2.15-9.69)	<0.001
Treating animals	No	1,460 (99.5)	1,431 (99.4)	29 (100.0)		
	Yes	8 (0.5)	8 (0.6)	0 (0.0)	-	-
Proximity to wild animals	No	899 (61.2)	889 (61.8)	10 (34.5)		
	Yes	569 (38.8)	550 (38.2)	19 (65.5)	3.07 (1.45-6.92)	0.004
Unusual illness humans	No	1,442 (98.2)	1,415 (98.3)	27 (93.1)		
	Yes	26 (1.8)	24 (1.7)	2 (6.9)	4.36 (0.68-15.77)	0.053
Unexplained human deaths	No	1,457 (99.2)	1,428 (99.2)	29 (100.0)		
	Yes	11 (0.8)	11 (0.8)	0 (0.0)	-	-
Abortion in herds	No	1,346 (91.7)	1,325 (92.1)	21 (72.4)		
	Yes	122 (8.3)	114 (7.9)	8 (27.6)	4.43 (1.81-9.86)	<0.001
Unexplained deaths in herds	No	1,310 (89.2)	1,289 (89.6)	21 (72.4)		
	Yes	158 (10.8)	150 (10.4)	8 (27.6)	3.27 (1.34-7.25)	0.005
Unexplained deaths	No	1,454 (99.0)	1,426 (99.1)	28 (96.5)		
in wild animals	Yes	14 (1.0)	13 (0.9)	1 (3.5)	3.92 (0.21-20.7)	0.2
Slaughtering dead animals	No	1,452 (98.9)	1,424 (99.0)	28 (96.5)		
	Yes	16 (1.1)	15 (1.0)	1 (3.5)	3.39 (0.18-17.65)	0.3
Eat bushmeat	No	1,376 (93.7)	1,351 (93.9)	25 (86.2)		

	Yes	92 (6.3)	88 (6.1)	4 (13.8)	2.46 (0.71-6.50)	0.102
Drink raw milk	No	1,448 (98.6)	1,420 (98.7)	28 (96.5)		
	Yes	20 (1.4)	19 (1.3)	1 (3.5)	2.67 (0.15-13.59)	0.4
Mosquito bites	No	372 (25.3)	366 (25.4)	6 (20.7)		
	Yes	1,096 (74.7)	1,073 (74.6)	23 (79.3)	1.31 (0.56-3.57)	0.6
Mosquito prevention	No	991 (67.5)	967 (67.2)	24 982.8)		
	Yes	477 (32.5)	472 (32.8)	5 (17.2)	0.43 (0.14-1.04)	0.085
Mosquito presence in home area	No	115 (7.8)	113 (97.8)	2 (6.9)		
	Yes	1,353 (92.2)	1,326 (92.2)	27 (93.1)	1.15 (0.34-7.20)	0.8
Mosquito increase in home area*	No	799 (54.4)	783 (54.4)	16 (55.2)		
	Yes	669 (45.6)	656 (45.6)	13 (44.8)	0.97 (0.45-2.03)	0.9
Proximity to swamp**	No	693 (47.2)	687 (47.7)	6 (20.7)		
	Yes	775 (52.8)	752 (52.3)	23 (79.3)	3.50 (1.51-9.53)	0.007

Legend: CI, confidence Interval; cOR, crude Odds Ratio.

*In the last 2 months, **within 20 km radius of home area
