# Supplementary material for: Understanding the Rift Valley fever exposure risk: A comparative perspective from a multi-country study in East and Central Africa, 2021-24
Source: PLoS Negl Trop Dis. 2026 Mar 10;20(3):e0014082. doi: 10.1371/journal.pntd.0014082 (PMC12987580; doi:10.1371/journal.pntd.0014082)
Supplement: S4 Table — Legend: CI, confidence Interval; cOR, crude Odds Ratio. *In the last 2 months, **within 20 km radius of home area. (PDF) [file pntd.0014082.s005.pdf]

S4 Table. Bivariate analysis by Rift Valley fever positivity for the cohort from Uganda.

Variable		Overall	Negative	Positive	cOR (95% CI)	p-value
Age-group	10-20 years old	427 (21.7)	401 (22.7)	26 (12.7)		
	21-40 years old	1.040 (52.8)	929 (52.7)	111(54.1)	1.84 (1.20-2.92)	0.007
	Above 40 years old	501 (25.5)	433 (24.6)	68 (33.2)	2.42 (1.53-3.95)	<0.001
Gender	Female	1,090 (55.4)	993 (56.3)	97 (47.3)		
	Male	878 (44.6)	770 (43.7)	108 (52.7)	1.44 (1.07-1.92)	0.014
Education	High Schooling	504 (25.6)	459 (26.0)	45 (21.9)		
	Low Schooling	1,464 (74.4)	1,304 (74.0)	160 (78.1)	1.25 (0.89-1.79)	0.2
Healthcare worker	No	1,900 (96.5)	1,697 (96.3)	203 (99.0)		
	Yes	68 (3.5)	66 (3.7)	2 (1.0)	0.25 (0.04-0.82)	0.057
Farmer (animal husbandry)	No	1,666 (84.7)	1,500 (85.1)	166 (81.0)		
	Yes	302 (15.3)	263 (14.9)	39 (19.0)	1.34 (0.91-1.93)	0.124
Farmer (crop cultivation)	No	772 (39.2)	707 (40.1)	65 (31.7)		
	Yes	1,196 (60.8)	1,056 (59.9)	140 (68.3)	1.44 (1.06-1.97)	0.020
Butcher	No	1,953 (99.2)	1,752 (99.4)	201 (98.1)		
	Yes	15 (0.8)	11 (0.6)	4 (1.9)	3.17 (0.87-9.36)	0.05
Similar disease in family*	No	1,585 (80.5)	1,421 (80.6)	164 (80.0)		
	Yes	383 (19.5)	342 (19.4)	41 (20.0)	1.04 (0.71-1.48)	0.8
Similar disease in community*	No	1,700 (86.4)	1,518 (86.1)	182 (88.8)		
	Yes	268 (13.6)	245 (13.9)	23 (11.2)	0.78 (0.48-1.21)	0.3
Keeping cattle	No	1,112 (56.5)	1,002 (56.8)	110 (53.7)		
	Yes	856 (43.5)	761 (43.2)	95 (46.3)	1.14 (0.85-1.52)	0.4
Keeping sheep	No	1,361 (69.2)	1,224 (69.4)	137 (66.8)		
	Yes	607 (30.8)	539 (30.6)	68 (33.2)	1.13 (0.82-1.53)	0.4
Keeping goat	No	842 (42.8)	763 (43.3)	79 (38.5)		
	Yes	1,126 (57.2)	1,000 (56.7)	126 (61.5)	1.22 (0.91-1.64)	0.2
Contact with cattle	No	1,184 (60.2)	1,070 (60.7)	114 (55.6)		
	Yes	784 (39.8)	693 (39.3)	91 (44.4)	1.23 (0.92-1.65)	0.2
Contact with sheep	No	1,502 (76.3)	1,358 (77.0)	144 (70.2)		
	Yes	466 (23.7)	405 (23.0)	61 (29.7)	1.42 (1.02-1.95)	0.031
Contact with goat	No	842 (42.8)	769 (43.6)	73 (35.6)		
	Yes	1,126 (57.2)	994 (56.4)	132 (64.4)	1.40 (1.04-1.90)	0.029
Herding animals	No	1,393 (70.8)	1,247 (70.7)	146 (71.2)		
	Yes	575 (29.2)	516 (29.3)	59 (28.8)	1.00 (0.71-1.34)	0.9
Milking animals	No	1,819 (92.4)	1,632 (92.6)	187 (91.2)		
	Yes	149 (7.6)	131 (7.4)	18 (8.8)	1.20 (0.69-1.96)	0.5
Assisting animal birthing	No	1,811 (92.0)	1,629 (92.4)	182 (88.8)		
	Yes	157 (7.8)	134 (7.6)	23 (11.2)	1.54 (0.94-2.41)	0.072
Slaughtering/skinning/	No	1,779 (90.4)	1,595 (90.5)	184 (89.8)		
butchering animals	Yes	189 (9.6	168 (9.5)	21 (10.2)	1.08 (0.65-1.71)	0.7
Handling raw meat	No	849 (43.1)	754 (42.8)	95 (46.3)		
	Yes	1,119 (56.9)	1,009 (57.2)	110 (53.7)	0.86 (0.65-1.16)	0.3
Cleaning animal areas	No	994 (50.5)	895 (50.8)	99 (48.3)		
	Yes	974 (49.5)	868 (49.2)	106 (51.7)	1.10 (0.83-1.48)	0.5
Feeding the animals	No	953 (48.4)	867 (49.2)	86 (41.9)		
	Yes	1,015 (51.6)	896 (50.8)	119 (58.1)	1.34 (1.00-1.80)	0.051
Sleeping with animals	No	1,896 (96.3)	1,697 (96.3)	199 (97.1)		
	Yes	72 (3.7)	66 (3.7)	6 (2.9)	0.78 (0.30-1.67)	0.6
Spraying animals	No	1,831 (93.0)	1,642 (93.1)	189 (92.2)		
	Yes	137 (7.0)	121 (6.9)	16 (7.8)	1.15 (0.64-1.92)	0.6
Treating animals	No	1,832 (93.1)	1,641 (93.1)	191 (93.2)		
	Yes	136 (6.9)	122 (6.9)	14 (6.8)	0.99 (0.53-1.69)	1.0
Proximity to wild animals	No	1,589 (80.7)	1,423 (80.7)	166 (81.0)		
	Yes	379 (19.3)	340 (19.3)	39 (19.0)	0.98 (0.67-1.41)	0.9
Unusual illness in humans	No	1,727 (87.7)	1,546 (87.7)	181 (88.3)		
	Yes	241 (12.3)	217 (12.3)	24 (11.7)	0.94 (0.59-1.45)	0.8
Unexplained human deaths	No	1,766 (89.7)	1,588 (90.1)	178 (86.8)		
	Yes	202 (10.3)	175 (9.9)	27 (13.2)	1.38 (0.87-2.09)	0.149
Abortion in herds	No	1,790 (90.9)	1,603 (90.9)	187 (91.2)		
	Yes	178 (9.1)	160 (9.1)	18 (8.8)	0.96 (0.56-1.56)	0.9
Unexplained deaths in herds	No	1,659 (84.3)	1,490 (84.5)	169 (82.4)		
	Yes	309 (15.7)	273 (15.5)	36 (17.6)	1.16 (0.78-1.68)	0.4
Unexplained deaths	No	1,952 (99.2)	1,750 (99.3)	202 (98.5)		
in wild animals	Yes	16 (0.8)	13 (0.7)	3 (0.7)	2.00 (0.46-6.26)	0.3
Slaughtering dead animals	No	1,926 (97.9)	1,726 (97.9)	200 (97.6)		
	Yes	42 (2.1)	37 (2.1)	5 (2.4)	1.17 (0.40-2.74)	0.7
Eat bushmeat	No	1,894 (92.2)	1,699 (96.4)	195 (95.1)		

	Yes	74 (3.8)	64 (3.6)	10 (4.9)	1.36 (0.65-2.58)	0.4
Drink raw milk	No	1,821 (92.5)	1,633 (92.6)	188 (91.7)		
	Yes	147 (7.5)	130 (7.4)	17 (8.3)	1.14 (0.65-1.87)	0.6
Mosquito bites	No	136 (6.9)	119 (6.7)	17 (8.3)		
	Yes	1,832 (93.1)	1,644 (93.3)	188 (91.7)	0.80 (0.48-1.41)	0.4
Mosquito prevention	No	477 (24.2)	426 (24.2)	51 (24.9)		
	Yes	1,491 (75.8)	1,337 (75.8)	154 (75.1)	0.96 (0.69-1.36)	0.8
Mosquito presence in home area	No	139 (7.1)	122 (6.9)	17 (8.3)		
	Yes	1,829 (92.9)	1,641 (93.1)	188 (91.7)	0.82 (0.50-1.44)	0.5
Mosquito increase in home area*	No	649 (33.0)	584 (33.1)	65 (31.2)		
	Yes	1,319 (67.0)	1,179 (66.9)	140 68.3)	1.07 (0.78-1.46)	0.7
Proximity swamp**	No	648 (32.9)	583 (33.1)	65 (31.7)		
	Yes	1,320 (67.1)	1,180 (66.9)	140 (68.3)	1.06 (0.78-1.46)	0.7

Legend: CI, confidence Interval; cOR, crude Odds Ratio.

*In the last 2 months, **within 20 km radius of home area
